# Supplementary material for: Machine learning with textural analysis of longitudinal multiparametric MRI and molecular subtypes accurately predicts pathologic complete response in patients with invasive breast cancer
Source: PLoS One. 2023 Jan 17;18(1):e0280320. doi: 10.1371/journal.pone.0280320 (PMC9844845; doi:10.1371/journal.pone.0280320)
Supplement: Attachment — Submitted filename: (FINAL) Response to reviewers.docx [file pone.0280320.s001.docx]

PONE-D-22-10717

Dear Editors and Reviewers,

We thank you for your thoughtful reviews of our manuscript. The point by point response and revised manuscript are attached. Thank you.

Tim Duong on behalf of coauthors.

Machine learning with textural analysis of longitudinal multiparametric MRI and molecular subtypes accurately predicts Pathologic Complete Response in patients with invasive breast cancer

PLOS ONE

ACADEMIC EDITOR:

This is a potentially interesting paper. However, the Authors should adequately address all the critical comments raised by the Reviewers. Moreover, they should discuss in greater detail the clinical impact of their findings. 

**Thank you. The followings have been added:**

**With further development and testing on larger multi-institutional sample sizes, this approach has the potential to non-invasively identify patients who are likely to respond to neoadjuvant chemotherapy at diagnosis or early treatment. This approach may prove useful for treatment planning, treatment execution, and mid-treatment adjustment to achieve better outcomes.**

Reviewers' comments:

Reviewer's Responses to Questions

**Comments to the Author**

1. Is the manuscript technically sound, and do the data support the conclusions?

The manuscript must describe a technically sound piece of scientific research with data that supports the conclusions. Experiments must have been conducted rigorously, with appropriate controls, replication, and sample sizes. The conclusions must be drawn appropriately based on the data presented.

Reviewer #1: Partly

Reviewer #2: Yes

2. Has the statistical analysis been performed appropriately and rigorously?

Reviewer #1: I Don't Know

**We expanded the section on Methods to better describe the statistical tests used. We also include statistical tests used in the results and legends where appropriate or unclear.**

Reviewer #2: Yes

3. Have the authors made all data underlying the findings in their manuscript fully available?

The [PLOS Data policy](https://nam04.safelinks.protection.outlook.com/?url=http%3A%2F%2Fwww.plosone.org%2Fstatic%2Fpolicies.action%23sharing&data=05%7C01%7Ctim.duong%40einsteinmed.edu%7C20ec094a61394fcfb37308da6e546471%7C9c01f0fd65e040c089a82dfd51e62025%7C0%7C0%7C637943607261808442%7CUnknown%7CTWFpbGZsb3d8eyJWIjoiMC4wLjAwMDAiLCJQIjoiV2luMzIiLCJBTiI6Ik1haWwiLCJXVCI6Mn0%3D%7C3000%7C%7C%7C&sdata=L2iB9xinfWvQm4bwe9igD5Z6821jq9ry9YAed2TsQH8%3D&reserved=0) requires authors to make all data underlying the findings described in their manuscript fully available without restriction, with rare exception (please refer to the Data Availability Statement in the manuscript PDF file). The data should be provided as part of the manuscript or its supporting information, or deposited to a public repository. For example, in addition to summary statistics, the data points behind means, medians and variance measures should be available. If there are restrictions on publicly sharing data—e.g. participant privacy or use of data from a third party—those must be specified.

Reviewer #1: Yes

Reviewer #2: Yes

4. Is the manuscript presented in an intelligible fashion and written in standard English?

PLOS ONE does not copyedit accepted manuscripts, so the language in submitted articles must be clear, correct, and unambiguous. Any typographical or grammatical errors should be corrected at revision, so please note any specific errors here.

Reviewer #1: Yes

Reviewer #2: Yes

5. Review Comments to the Author

Please use the space provided to explain your answers to the questions above. You may also include additional comments for the author, including concerns about dual publication, research ethics, or publication ethics. (Please upload your review as an attachment if it exceeds 20,000 characters)

Reviewer #1: 1) Given that DTI is a truly quantitative technique, a quality control of scanner performances in general and of correct application of diffusion weighting gradients in particular is needed, in order to guaranty accurate estimates of diffusion indices and reproducible results in clinical studies (Jones, Top Magn Reson Imaging 2010, 21: 87-99). Moreover, this is of paramount importance when pooling data from different scanners. Indeed, previous large multicenter studies have shown a non-negligible variability in quantitative diffusion indices measured using different scanner systems (Fedeli et al, Phys Med 2018, 55: 135-141; Fedeli et al, Phys Med 2021, 85: 98-106). Therefore, MR scanner system should be adequately characterized in diffusion-MRI of the breast (Giannelli et al, PLoS One 2014, 9: e86280). The Authors should discuss in detail these aspects.

**We completely agree with these comments. Raw images are not available to make corrections. Per open data sources, these images underwent reasonable and vigorous data quality check, , ranging from distortion correction, eddy current correction, quality assurance with phantom and human data, pooling data from multiple sites. Thank you for the references. They are cited. The following is added:**

**We only performed visual check on images as provided but did not perform distortion correction, eddy current correction, check for multisite scanner consistency, among others, as raw data were not available. It has been shown that accurate estimates and reproducibility of diffusion indices in clinical studies require careful data quality assurance (Jones, Top Magn Reson Imaging 2010, 21: 87-99). Moreover, large multicenter studies have shown a non-negligible variability in quantitative diffusion indices measured using different scanner systems (Fedeli et al, Phys Med 2018, 55: 135-141; Fedeli et al, Phys Med 2021, 85: 98-106). Thus, MRI scanner system should be adequately characterized in diffusion-MRI of the breast (Giannelli et al, PLoS One 2014, 9: e86280).**

2) The Authors have focused on a single feature class, i.e., GLCM. The Authors should explain this choice and why they have not considered additional feature classes.

**Thank you for your comment. The following is added.**

**While there are many texture features have been explored, we only chose GLCM features because they haven't been widely used before compared to other radiomic features. Future studies should compare results from different texture features.**

3) When applying machine learning techniques on multicenter data, it would be appropriate using methods of data harmonization (Johnson et al, Biostatistics 2007, 8: 118-127). The Authors should hence discuss this limitation of their study.

**Thank you for the references. They are cited. The following is added:**

**When applying machine learning techniques on multicenter data, data harmonization (Johnson et al, Biostatistics 2007, 8: 118-127) may be necessary. However, we did not perform additional data harmonization as all MRI acquisitions were taken with ISPY2 acquisition requirements as described in (20). Furthermore, we ensured that data from different field strengths and different molecular subtypes, etc., were not over-represented in either training and testing data sets by using 5-fold cross validation so that all data has the chance to be in both the test and training sets for a given fold.**

4) Given the sensitivity of radiomic features to image resampling and discretization, the Authors should report the resampling voxel size and bin width/number of bins used for the preprocessing of radiomic data, discussing whether the used values are optimal.

**Thank you for the comment. The following is added:**

**All images were interpolated to 0.7825 mm x 0.7825 mm voxel spacing with a 2.5 mm slice thickness, which was the median spacing and thickness for all patients. Images were cast to 8-bits, and the number of bins was set to the maximum value of 256 to maximize the number of grey levels counted.**

Reviewer #2: In this retrospective paper, the authors were able to predict pCR after neoadjuvant chemotherapy using XGBoost with MRI and non-imaging data at multiple treatment timepoints.

**For all comments below, all were revised as suggested (but not marked) except where details are noted:**

1. In Abstract Purpose and throughout the paper, I suggest pathological complete response (pCR) and extreme gradient boosting (XGBoost).
2. In Abstract Results, 1st sentence, was 0.903 an AUC value?
3. In Introduction, 1st paragraph, last sentence, more details are needed.

**It is revised as:**

**Thus, accurate prediction of pCR could help treatment planning, identifying patients who are likely to respond to specific NAC drugs , and alter treatments mid-course if needed, to maximize successful outcomes while minimize adverse effects of unnecessary chemotherapy.**

4. In Introduction, 2nd paragraph, 1st sentence, a should be added before chemotherapy.
5. In Introduction, 3rd paragraph, 2nd sentence, see reviews can be cut and and should be added before DCE dynamics.
6. In Methodology, Data Sources, 1st paragraph, penultimate sentence, I suggest logistic regression.
7. In Methodology, Data Sources, 2nd paragraph, 1st sentence, and throughout the paper, I suggest paclitaxel and anthracycline.
8. In Methodology, Ground Truth, 1st sentence, FTV is not clear.
9. In Methodology, Statistical Analysis, 3rd paragraph, line 2, Recall should be recall. In line 4, the material in parentheses, starting with model, can be deleted.
10. In Results, 1st paragraph, 2nd sentence, the should be added before mean.
11. In Discussion, 4th paragraph, line 8, derived from should be cut.
12. In Conclusion, 1st sentence, subtypes should be changed to subtype. In the 2nd sentence, a should be added after predict.
13. The references should be written according to the journal's style.
14. In the figures, I suggest one patient per figure, and the pulse sequences should be described in the legends

**Thank you. all are revised as suggested.**
