# Supplementary material for: Machine learning with textural analysis of longitudinal multiparametric MRI and molecular subtypes accurately predicts pathologic complete response in patients with invasive breast cancer
Source: PLoS One. 2023 Jan 17;18(1):e0280320. doi: 10.1371/journal.pone.0280320 (PMC9844845; doi:10.1371/journal.pone.0280320)
Supplement: Attachment — Submitted filename: Response.docx [file pone.0280320.s002.docx]

R3

**Dear Dr. Giannelli and reviewers,**

**Thank you for your careful reading of the manuscript.**

**Thank you for the grammatic corrections.**

**Please find the point-by-point responses and the revised manuscript.**

**I wish you and your family a happy holiday season.**

**Sincerely, Tim Duong.**

Reviewer #2: This paper on assessing response to neoadjuvant chemotherapy in breast cancer with MRI has been improved since an earlier submission.
1. In the 1st paragraph of the Introduction, 2nd sentence, I suggest: Pathological complete response...tumor present at surgery post NAC.
2. In the fourth paragraph of the Introduction, the 2nd sentence should start with Extreme gradient boosting.
3. In Materials and Methods, Data Sources, last sentence of the last paragraph, I suggest: data had the...test and the training sets.
4. In Materials and Methods, Ground Truth, first sentence, I suggest: functional tumor volume.
5. In Results, Prediction of pCR, last paragraph, 2nd sentence, model should be models.
6. In Discussion, 4th paragraph, 6th sentence, from should be deleted.
7. In Limitations, 4th sentence, image should be images.
8. The references should be written according to the journal's style. The journal names should be capitalized and abbreviations should be used. In reference 16, in the title of the chapter, rrimary should be primary, and the title of the book should start with Breast. In reference 19, Association should be association.

**Thank you for all the grammatic corrections above. Changes are not tracked.**

9. I suggest one patient per figure. In the figure legends, I suggest including the age of the patient and the breast tumor pathologic diagnosis.

**This information is provided as suggested.**

Fig 3. Post-contrast MRIs from DCE for (A) a pCR patient and (B) a non-PCR patient at the pre-treatment, early treatment, and mid-treatment time points. For (A), the 54 year old Caucasian patient had invasive ductal carcinoma with HR- and HER2-. For (B), the 51 year old Caucasian patient had invasive ductal carcinoma with HR+ and HER2-. The yellow arrow indicates the approximate location of the tumor. MRI images were taken approx. 2.5 minutes after contrast injection with a phase duration of 80sec < phase duration < 100sec. pCR: complete pathological response, DCE: dynamic contrast enhanced.

Fig 4. DWIs for (A) a pCR patient and (B) a non-PCR patient at the pre-treatment, early treatment, and mid-treatment time points. For (A), the 54 year old Caucasian patient had invasive ductal carcinoma with HR- and HER2-. For (B), the 51 year old Caucasian patient had invasive ductal carcinoma with HR+ and negative HER2-. The yellow arrow indicates the approximate location of the tumor. DWI acquisitions were done using a b-value of 800 s/mm^2^. DWI: diffusion-weighted imaging).
